# Elevated C-Reactive Protein in Children from Risky Neighborhoods: Evidence for a Stress Pathway Linking Neighborhoods and Inflammation in Children

**DOI:** 10.1371/journal.pone.0045419

**Published:** 2012-09-25

**Authors:** Stephanie T. Broyles, Amanda E. Staiano, Kathryn T. Drazba, Alok K. Gupta, Melinda Sothern, Peter T. Katzmarzyk

**Affiliations:** 1 Pennington Biomedical Research Center, Louisiana State University System, Baton Rouge, Louisiana, United States of America; 2 School of Public Health, Louisiana State University Health Sciences Center, New Orleans, Louisiana, United States of America; German Diabetes Center, Leibniz Center for Diabetes Research at Heinrich Heine University Duesseldorf, Germany

## Abstract

**Background:**

Childhood socioeconomic status is linked to adult cardiovascular disease and disease risk. One proposed pathway involves inflammation due to exposure to a stress-inducing neighborhood environment. Whether CRP, a marker of systemic inflammation, is associated with stressful neighborhood conditions among children is unknown.

**Methods and Results:**

The sample included 385 children 5–18 years of age from 255 households and 101 census tracts. Multilevel logistic regression analyses compared children and adolescents with CRP levels >3 mg/L to those with levels ≤3 mg/L across neighborhood environments. Among children living in neighborhoods (census tracts) in the upper tertile of poverty or crime, 18.6% had elevated CRP levels, in contrast to 7.9% of children living in neighborhoods with lower levels of poverty and crime. Children from neighborhoods with the highest levels of either crime or poverty had 2.7 (95% CI: 1.2–6.2) times the odds of having elevated CRP levels when compared to children from other neighborhoods, independent of adiposity, demographic and behavioral differences.

**Conclusions:**

Children living in neighborhoods with high levels of poverty or crime had elevated CRP levels compared to children from other neighborhoods. This result is consistent with a psychosocial pathway favoring early development of cardiovascular risk that involves chronic stress from exposure to socially- and physically-disordered neighborhoods characteristic of poverty.

## Introduction

In adults, low socioeconomic status (SES) has been consistently linked to higher cardiovascular disease (CVD) risk. [1], [2] Similarly, growing evidence links low SES to prevalence of the metabolic syndrome (MetS) [3], [4], [5], [6], [7] and incidence of Type II diabetes (diabetes), [8], [9], [10], [11], [12] risk factors for CVD. Low SES may influence disease risk through behavioral pathways involving poor diet, [13] physical inactivity, [14] and smoking, [15] which often begin in childhood. These behaviors are also associated with low childhood SES. [16], [17] Thus, it is not surprising that low childhood SES has also been implicated in the development of adult metabolic syndrome [7], diabetes [10], and CVD [18], [19].

As an alternative to behavioral pathways, stress has been proposed as a link between poverty and CVD risk. [20], [21], [22] Chronic stress promotes dysregulation of the autonomic nervous system and the hypothalamic-pituitary-adrenal axis, and the resulting inflammation is linked to obesity and the metabolic syndrome. [23], [24], [25], [26] Poverty is often characterized by more frequent exposures to psychosocial stress, including living in socially- and physically-disordered environments. [20], [27], [28], [29], [30] Therefore, exposure to a stressful neighborhood environment may be an important aspect of poverty’s influence on CVD risk. Consistent with this pathway, studies in adults have noted relationships between obesity, [31] diabetes incidence, [32] and heart disease [33], [34] with negative aspects of the neighborhood environment (negative perceptions, [32] neighborhood unemployment and crime, [33], and an index of neighborhood psychosocial stress that includes area SES in its measure [31], [34]).

C-reactive protein (CRP) is a marker of systemic inflammation, and increases in levels of CRP are thought to be part of the cascade of biological responses to chronic stress. [25], [35], [36], [37]. Elevated serum CRP has been found in adults with prediabetes, prehypertension, obesity, diabetes, hypertension and CVD. [38], [39], [40], [41], [42] Childhood CRP levels correlate with CVD risk factors [43], [44] and track into adulthood. [45].

Several studies in adults have noted significant relationships between CRP and neighborhood SES. [46], [47], [48], [49] A growing body of literature has documented links between childhood adversity (measured at the individual or household level) and inflammation; [50] however, whether *neighborhood* sources of stress are linked to inflammation or other markers of early CVD risk in children has not been well-studied. To address this gap, we examined whether children living in neighborhoods having high levels of poverty or crime (i.e., higher exposure to neighborhood sources of psychosocial stress) had higher levels of CRP than children living in neighborhoods with lower levels of poverty or crime.

## Methods

### Ethics Statement

All study procedures were approved by the Pennington Biomedical Research Center institutional review board, and the parents of the participants provided signed informed consent, with children providing verbal assent.

### Sample

Participants included children aged 5–18 years recruited for an assessment of factors related to abdominal adiposity. Recruitment occurred through study advertisements (television and print) targeting the Baton Rouge, Louisiana metropolitan area, as well as through pediatricians’ offices. Recruitment attempted to balance the sample across race, sex, and body mass index (BMI) categories over the course of the study. Four-hundred twenty-three (423) children and adolescents participated in the study, which enrolled participants from February 2010 through August 2011.

Of the 423 participants, 9 provided address information that could not be geocoded (e.g., post office boxes). Of the remaining 414 participants, 14 refused to provide a blood sample and, therefore, were missing information on the outcome variable. Participants with CRP>10 mg/L, indicating current or recent acute infection (n = 15), were excluded in order to ensure that the elevated CRP was of non-infectious origin. [51] The final analytic sample consisted of 385 children.

### Biological Measures

Fasting serum CRP levels were measured with a high-sensitivity chemiluminescent immunoassay (Siemens Immulite 2000; Deerfield, IL); the lower limit of detection was 0.20 mg/L (37.7% of the sample). The interassay coefficient of variation was 3.7%. Body fat percentage was measured by dual-energy x-ray absorptiometry (DXA) (Hologic QDR 4500A; Bedford, MA).

### Neighborhood Measures

Participant addresses were geocoded to the census tract level and linked to census-tract family poverty (US Census 2000) and an index of total crime derived from Uniform Crime Report data (CrimeRisk, Applied Geographic Solutions, 2010). The 2010 CrimeRisk index included data for the years 1998–2006, supplemented with preliminary 2007 release data. The index is adjusted for population and scaled to be relative to the national index of 100. For example, a CrimeRisk index of 150 indicates a crime risk 150% the national average. Within the sample, census tract poverty and crime levels were categorized into tertiles (low, medium, and high).

### Other Covariates

A study questionnaire was used to obtain self-reported data on additional covariates. Children completed the survey, with the help of the accompanying parent when necessary. For children under the age of 10, the accompanying parent generally completed the survey.

#### Household socioeconomic status

To address the fact that measures of household socioeconomic status were highly correlated with each other and with race, a single household socioeconomic status factor was created using principal components analysis, controlling for race. This factor (Cronbach’s α = 0.70) combined the effects of household poverty income ratio (PIR), father’s educational attainment, and mother’s educational attainment. PIR was created based on the 2009 Federal Poverty Threshold, [52] using self-reported household income (8 categories; $20,000 increments from <$10,000 to >$140,000) and household size. For the household income category that was reported, the median value within the category was used as the value of household income in calculating PIR; for the two extreme categories, the threshold values of $10,000 or $140,000 were used. Father and mother educational attainment was self-reported as grades 0–8, some high school, high school diploma/GED, 1–3 years college, college degree, or post graduate degree.

#### Self-reported diet and physical activity

Children reported on levels of physical activity based on a physical activity screening question (days in the past week with at least 60 minutes of physical activity that increased your heart rate and made you breathe hard some of the time) [53] used by both the US Youth Risk Behavior Survey (YRBS) and the Health Behavior in School-Aged Children (HSBC) survey. Children reported on usual intake of various food items, with response options of never, less than once a week, once a week, 2–4 days a week, 5–6 days a week, once a day/every day, or every day more than once. Measures of fruit and vegetable consumption (daily consumption versus less), sweets/sugar-sweetened beverage (SSB) consumption (consumed <2 days per week versus more), and fish consumption (consumed ≥2 days per week versus less) were summed to create an index of a healthy dietary pattern, based on one used to track attainment of the American Heart Association’s criteria for ideal cardiovascular health. [54].

### Treatment of Missing Data

Thirty-five participants (9.1%) were missing data on adiposity (n = 4), a household socioeconomic status measure (n = 28), or a behavioral measure (n = 3). Each variable was missing <5%; father’s educational attainment was missing most often, at 4.9%. Participants missing data for any of the covariates were similar to those with complete covariate data with respect to CRP level, race, sex, age, household SES, adiposity, and neighborhood characteristics.

Missing values were multiply-imputed (5 imputations) for these participants using Markov chain Monte Carlo (MCMC) methods, which accommodates non-monotone missing data patterns, under missing at random (MAR) assumptions [55] and using SAS version 9.3 (PROC MI). The imputation model contained all variables included in the full model (Model 3), with the addition of height, which improved imputation of body fat percentage. Although all variables were not normally-distributed, MCMC multiple imputation is robust to departures from normality when the amount of missing data for a particular variable is small. [56] Results across the five imputed datasets were averaged, and the standard errors were adjusted appropriately, using the MIANALYZE procedure in SAS. Sensitivity analyses also examined results from analyses of participants with complete data, and results were similar.

### Analysis

A series of multilevel, multivariable logistic regression analyses (SAS version 9.3, PROC GLIMMIX) was used to examine the relationship between the neighborhood environment and elevated CRP. Because of the large proportion (37.7%) of participants at the lower detection limit, a linear regression model was inappropriate; consequently, CRP was dichotomized as >3 mg/L to those with levels ≤3 mg/L, consistent with other research in children [57], [58] and the definition of “high risk” in adults. [59] Models accounted for both family and neighborhood clustering and compared children and adolescents with CRP levels >3 mg/L to those with levels ≤3 mg/L. Model 1 included the covariates of race (African American versus non-African American), sex, age, and household SES. Because CRP levels are strongly associated with adiposity in children, [60], [61], [62], [63] Model 2 also included a direct measure of adiposity, percent body fat. Model 3 contained the covariates from Model 2, with the addition of physical activity and diet. Each series of models was run according to the following specificationss of neighborhood crime and poverty: I) neighborhood poverty (continuous, centered, standardized) alone in a model, II) neighborhood crime (continuous, centered, standardized) alone in a model, III) neighborhood poverty and crime (continuous, centered, standardized) and their interaction, IV) neighborhood poverty at each tertile of crime, V) neighborhood crime at each tertile of poverty, and VI) neighborhood poverty and crime dichotomized as high neighborhood crime or poverty versus low/medium crime and poverty.

Interaction effects between model covariates were tested where warranted (i.e., when main effects were significant); however, none were identified. Interaction effects were also used to test for sex- and race-based differences in the relationship between elevated CRP levels and the neighborhood environment. These relationships did not differ significantly by race or by sex in any of the models; therefore, overall results are presented.

## Results

**Figure 1:**
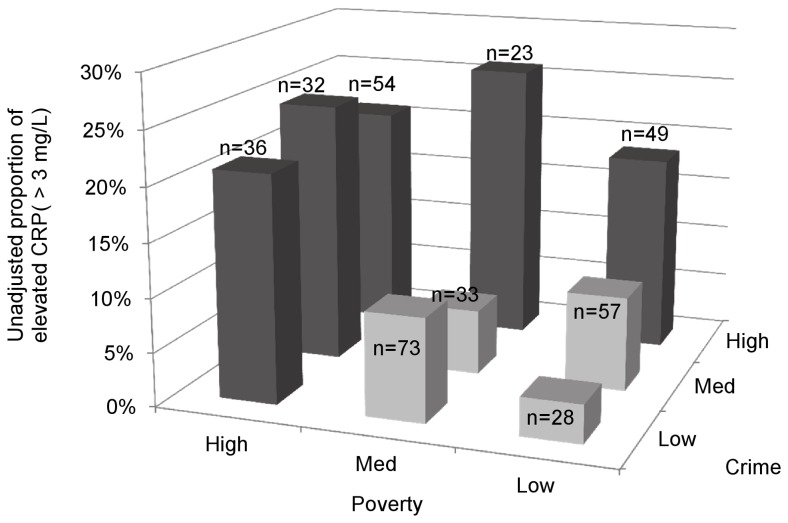
Variation in elevated C-reactive protein concentrations across neighborhood (census tract) poverty and crime.

After excluding observations with CRP levels indicative of a current or recent acute infection, 13.3% of children had CRP levels >3 mg/L. Among children living in neighborhoods (census tracts) with high levels of poverty or crime, 18.6% had elevated CRP levels, in contrast to 7.9% of children living in neighborhoods with lower (low or medium) levels of poverty and crime (Figure 1 and Table 1).

**Table 1:** Study participant characteristics by neighborhood environment.

Characteristic		High poverty or crime neighborhood(n = 194)^1^	Low/medium poverty & crime neighborhood(n = 191)^2^
Elevated CRP (>3 mg/L), n (%)		36 (19)	15 (8)
CRP (mg/L), median (IQR)		0.4 (<0.2–1.9)	0.3 (<0.2–0.9)
Race/ethnicity, n (%)	AA^3^	125 (64)	63 (33)
	White	65 (34)	119 (62)
	Other race	4 (2)	9 (5)
Sex, n (%)	Male	95 (49)	92 (48)
	Female	99 (51)	99 (52)
Age, mean (sd) [range]		11.6 (3.7) [5]–[18]	12.0 (3.4) [5]–[18]
BMI categories^4^, n (%)	Normal wt.	87 (45)	102 (53)
	Overweight	33 (17)	31 (16)
	Obese	74 (38)	58 (30)
BMI-z, mean (sd) [range]		1.1 (1.2) [−2.2–3.2]	0.9 (1.1) [−2.5–2.7]
Body Fat %, mean (sd) [range]		27.9 (10.1) [9.9–49.2]	27.6 (9.5) [9.9–46.8]
Body Fat (kg), mean (sd) [range]		16.3 (11.6) [2.9–61.8]	16.4 (10.8) [2.8–55.6]
Days of 60-min MVPA per week, mean (sd) [range]		3.2 (2.1) [0–7]	3.5 (2.1) [0–7]
Daily consumption of fruits & vegetables, n (%)		36 (19)	34 (18)
Consumption of fish ≥2 days/week, n (%)		43 (22)	28 (15)
Consumption of sweets/SSBs^5^≥2 days/week, n (%)		155 (80)	160 (84)
Mother completed college, n (%)		79 (41)	92 (48)
Father completed college, n (%)		57 (31)	77 (42)
Household poverty income ratio, n (%)	<130%	63 (33)	28 (15)
	130%–349%	76 (40)	69 (37)
	≥350%	53 (28)	89 (48)

1 194 participants from 131 households, living in 49 census tracts;

2 191 participants from 124 households, living in 52 census tracts;

3 AA = African American;

4 Normal weight (<85^th^ percentile for sex and age), overweight (≥85^th^ and <95^th^ percentile for sex and age), obese (≥85^th^ percentile for sex and age);

5 SSBs = sugar sweetened beverages.

Study participants represented 255 households from 101 census tracts, 75% of which were located in East Baton Rouge Parish (county), Louisiana. Across all census tracts represented in the sample, the percent of families living in poverty ranged from 0% to 44.2%, with a mean of 13.0% (Table 2). An index of total crime ranged from 6 to 454, with a mean of 202.3. In our sample, the cutoff for high poverty and high crime (the upper tertile within the sample) corresponded to levels of 16.4% and 279, respectively. Poverty and crime were correlated (r = 0.23, p<0.0001). However, when separated into low, medium, and high levels of poverty or crime based on tertiles within the sample, 34 (34%) census tracts had disparate levels of poverty and crime: 17 (17%) census tracts were considered high poverty but low or medium crime, and 17 (17%) were considered high crime but low or medium poverty.

**Table 2:** Characteristics of study participant neighborhoods (census tracts).

Characteristic	Overall(n = 101)	High poverty or crime neighborhood (n = 49)	Low/mediumpoverty & crime neighborhood (n = 52)
Percent of families in poverty, mean (SD) [range]	13.0 (10.5) [0–44.2]	18.9 (11.4) [0–44.2]	6.9 (4.0) [0–15.0]
Index of total crime, mean (SD) [range]	202.3 (125.0) [6–454]	281.1 (107.1) [0–44.2]	121.8 (84.3) [6–274]

Neither poverty nor crime was associated with elevated CRP when considered singly in a model (Table 3: Models I and II); however, these neighborhood effects interacted significantly (p = 0.02, Models III:1–3). In a model that included main effects for both neighborhood crime and poverty as well as their interaction, both neighborhood crime and poverty were positively associated with elevated CRP; however, as levels of crime or poverty increased, the effect of the other was dampened, as evidenced by an interaction effect <1.0. When poverty was considered within levels of crime (Table 3: Model IV), the effect of poverty on risk for elevated CRP was strongest in low crime areas, and the effect of poverty on CRP decreased significantly with increasing crime (p for trend <0.01 for Models IV:1–3). However, when crime was considered within levels of poverty (Table 3: Model V), the effect of crime on CRP was similar across all levels of poverty. When neighborhood poverty and crime were combined into a single dichotomous term (high neighborhood poverty or crime *vs.* low or medium poverty and crime), children from neighborhoods with the highest levels of either crime or poverty had 2.7 (95% CI: 1.2–6.2) times the odds of having high CRP levels when compared to children from other neighborhoods, independent of adiposity, demographic and behavioral differences.

**Table 3:** Odds ratios and 95% confidence intervals for elevated CRP (>3 mg/L) associated with neighborhood crime and poverty.

Neighborhood effects included in model	Effect	Model 1^1^	Model 2^2^	Model 3^3^
Model I. Poverty^4^	Poverty	1.17 (0.8–1.7)	0.98 (0.7–1.5)	0.98 (0.6–1.5)
Model II. Crime^5^	Crime	1.38 (1.0–1.9)	1.22 (0.8–1.8)	1.21 (0.8–1.8)
Model III. Poverty,^4^ crime,^5^ andpoverty-crime interaction	Poverty	1.66 (1–2.7)*	1.41 (0.8–2.4)	1.44 (0.8–2.5)
	Crime	1.24 (0.9–1.8)	1.1 (0.7–1.7)	1.09 (0.7–1.7)
	Poverty × crime	0.59 (0.4–0.9)*	0.55 (0.3–0.9)*	0.55 (0.3–0.9)*
Model IV. Poverty^4^ at each tertile ofneighborhood crime	Poverty within low crime	3.32 (1.3–8.4)*	2.49 (0.9–3.9)	2.63 (0.9–7.4)
	Poverty within medium crime	1.66 (0.7–3.8)	2.07 (0.7–5.7)	2.29 (0.9–6.1)
	Poverty within high crime	0.83 (0.6–1.2)	0.60 (0.4–1.0)	0.64 (0.4–1.1)
		p_trend_ = 0.0072	p_trend_ = 0.0157	p_trend_ = 0.0166
Model V. Crime^5^ at each tertile of neighborhood poverty	Crime within low poverty	1.76 (0.8–3.7)	1.70 (0.7–4.1)	1.71 (0.7–4.1)
	Crime within medium poverty	1.66 (1–2.8)*	1.59 (0.9–2.9)	1.57 (0.9–2.9)
	Crime within high poverty	0.80 (0.4–1.5)	0.52 (0.2–1.2)	0.50 (0.3–1.2)
		p_trend_ = 0.1150	p_trend_ = 0.0498	p_trend_ = 0.0479
Model VI. Poverty and crime combinedinto a single dichotomous term	High neighborhood crimeor poverty^6^	2.56 (1.3–5.2)**	2.66 (1.2–6.1)**	2.69 (1.2–6.2)**

1 Model 1 is adjusted for sex, race, age, and household SES.

2 Model 2 is adjusted for sex, race, age, household SES, and body fat percentage.

3 Model 3 is adjusted for sex, race, age, household SES, body fat percentage, physical activity level, and dietary pattern.

4 Continuous and standardized; odds ratio represents the increased odds of elevated CRP corresponding to a 1 SD increase in neighborhood poverty.

5 Continuous and standardized; odds ratio represents the increased odds of elevated CRP corresponding to a 1 SD increase in neighborhood crime.

6 Ref = Medium/low neighborhood crime and poverty.

* p<0.05, ** p<0.01.

Across all analytic specifications of neighborhood crime or poverty, inclusion of adiposity in the model (Model 2 vs. Model 1) generally attenuated the association between neighborhood crime/poverty and CRP. Further inclusion of behavioral variables (Model 3 vs. Model 2) did not appear to modify the estimated relationships.

## Discussion

In a cross-sectional sample of children ages 5 to 18 years, children from neighborhoods characterized by high levels of poverty or crime exhibited higher levels of CRP, a marker of systemic inflammation and cardiovascular risk that tracks into adulthood. [45] The life course model for disease development suggests that risk due to exposure to adverse environments accumulates over a person's lifetime, beginning in childhood. [18] Although considerable evidence links childhood SES with adult CVD, the mechanisms for this association remain poorly understood. Our findings suggest that inflammation due to exposure to a stress-inducing neighborhood environment may be one pathway.

A recent review noted that stress may be a factor on par with diet and physical activity in the development of obesity and related metabolic disease. [64] Our research suggests that children living in neighborhoods with high levels of poverty or crime may be at increased risk of inflammation resulting from exposure to stressful neighborhood conditions. This result is consistent with others in adults that noted significant relationships between CRP and neighborhood SES, [46], [47], [48], [49] between abnormal glucose metabolism and area SES, [65] and between heart disease and neighborhood unemployment and crime [33] or an index of neighborhood psychosocial stress. [34] More numerous are studies noting an association between individual-level measures of adversity, like household SES, and inflammation in children (reviewed in [50]) or between household SES and CRP in adults [49], [66], [67], [68], [69] or other biomarkers of CVD risk in adolescents. [70] However, results are mixed, and some associations are attenuated after adjustment for adiposity. [69], [71], [72], [73] Furthermore, recent work has noted an association between neighborhood disorder and serum cortisol levels in children, [74] providing additional support for a neighborhood stress-inflammation relationship in children.

**Figure 2:**
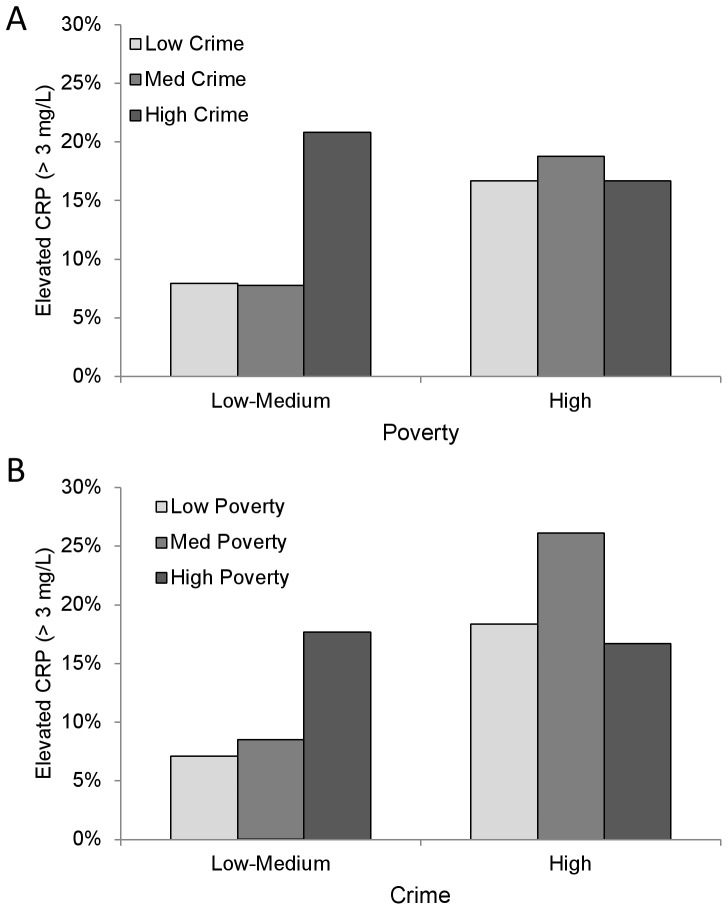
Covariate-adjusted elevated C-reactive protein (CRP) concentrations across neighborhood (census tract) poverty and crime levels. (A) Percent of children with elevated CRP in low, medium, and high crime neighborhoods, by low-medium poverty versus high poverty neighborhoods, and (B) percent of children with elevated CRP in low, medium, and high poverty neighborhoods, by low-medium crime versus high crime neighborhoods.

Results further suggest that neighborhood crime and poverty are not independent in their association with elevated CRP. Specifically, the effect of poverty was strongest at lower levels of crime and decreased with increasing crime, and the effect of crime was strongest at lower levels of poverty and decreased with increasing poverty. While neither crime nor poverty appeared to be associated with elevated CRP when considered individually in a model, associations were apparent when the analysis focused on participants living in the low and medium tertiles of the other neighborhood effect. Children living in the highest tertile of crime or poverty experienced the highest risk of elevated CRP; however, within these groups, exposure to an additional neighborhood stressor did not appear to alter risk (Figure 2). Consequently, children exposed to *either* high neighborhood crime *or* high neighborhood poverty appear to be at highest risk of elevated CRP. Studies of the influence of the neighborhood environment have cited the importance of “context” in understanding health and health behavior. [75] Our results further suggest that even when studying a neighborhood effect, the expanded context of the neighborhood may matter. Studies of neighborhood effects typically investigate effects singly, and our results suggest that important effects may be missed, or their effect sizes attenuated, by such an approach.

In the current study, the effect size seen for high poverty or crime neighborhood and elevated CRP is estimated to be 0.55, [76] which is considered a medium effect. [77] Neighborhood effects on children have typically been small; [78] although, there are examples of studies with similarly-sized effects. In a study looking at the relative contribution of neighborhood and household measures of SES, neighborhood income and education levels were stronger than their household equivalents in adjusted analyses of BMI and cortisol level. [79] Neighborhood SES independently accounted for 9.2–10.6% of the variance in BMI, equivalent to a moderate effect size. In another study that looked at area effects on fibrinogen, [80] the effect size noted, while apparently small for a unit change in the deprivation index used (0.05 SD per unit change), could approach a medium effect size if children from the upper and lower proportions of the neighborhood deprivation range (an absolute range of 15.1 units in the sample) had been compared. Lastly, a study of neighborhood SES and cardiovascular responsivity to and recovery from laboratory stressors found significant associations in both white and African American children. [81] Both heart rate responsivity and recovery showed medium effects between the upper and lower neighborhood SES groups in both white (−0.58 SD responsivity effect and −0.46 SD recovery effect) and African American children (0.52 SD responsivity effect and 0.62 SD recovery effect), although the direction of the effect differed between the two race groups.

While not the focus of this study, household SES was not related to elevated CRP in our analyses. Although research in adults has documented inverse relationships between markers of socioeconomic status and CRP levels, [67], [68] this relationship has been inconsistent in children. [50] Even among studies reporting associations, the associations generally disappear after adjustment for adiposity. [69], [72], [73] It is not entirely clear why inflammation in children would be more strongly related to neighborhood conditions, versus household SES, and we look to future studies to confirm our results. However, a study investigating the joint contributions of family and neighborhood SES on health markers in adolescents found that neighborhood SES was more strongly related to BMI and basal cortisol levels than family SES. [79] Other research reporting associations between area conditions and cortisol [74] or fibrinogen [80] did not adjust for household SES.

Studies of CRP in children have consistently documented a strong relationship between CRP and indirect measures of adiposity, [60], [61], [62], [63] with BMI or derivative measures (e.g., BMI percentiles or BMI categories) being the most common. In the current study, a direct measure of adiposity – body fat percentage, assessed by DXA – was used. DXA is considered to provide the most accurate body composition analysis in children. [82] While BMI is the most common metric for assessing adiposity, it in fact measures excess body mass, which can be composed of either lean or fat mass, and its accuracy in measuring adiposity varies according to a child’s actual adiposity (assessed by DXA). [83], [84] While not reported, among all covariates, body fat percentage showed the strongest association with elevated CRP across all models. Additionally, all results were consistent when sex- and age-specific BMI z-scores were substituted for body fat percentage in the analysis.

CRP levels become elevated after infection, and inverse relationships between SES and illness have been noted. [85] Moreover, elevated CRP levels are associated with obesity in children, [60], [61], [62], [63] which has also been linked to both individual/family and neighborhood SES. [86], [87], [88], [89] A particular strength of this study is its ability to focus on the effect of interest, i.e., the proposed pathway whereby the neighborhood environment promotes chronic stress and elevated CRP levels, as analyses were adjusted for a direct measure of adiposity and other confounding relationships. Disentangling neighborhood effects of SES on health is methodologically challenging. Our use of multilevel models that account for within-household similarities and shared environment, including psychosocial stressors in the household, strengthen the evidence for the neighborhood effect.

The cross-sectional study design limits our findings, as these results cannot demonstrate causative relationships. Furthermore, participants self-selected into the study, and recruitment attempted to balance across race, sex, and BMI categories; therefore, the participant sample cannot be considered demographically representative of the geographic area in which study recruitment occurred. Study participants did represent 76 (83%) of the census tracts within the parish (county) in which the majority of participants resided, however. Also, census tracts in which study participants resided were generally similar to those not represented in the sample.

Our results may also be limited by the presence of missing data. A sensitivity analysis did not reveal differences between analyses of complete cases and results reported here; however, neither complete case analysis nor multiple imputation can correct for the 23 participants excluded from the analysis because of missing exposure (address not able to be geocoded) or missing outcome (refusal of blood draw). Our results could be biased if these excluded participants differed in the adjusted relationship between their neighborhood conditions and CRP levels, although the effect is likely to be minimal given the small number of exclusions.

A large proportion of study participants (37.7%) had CRP levels at the lower limit of detection, precluding analysis of CRP as a continuous outcome, as has been done in other studies. Additionally, there are no specific pediatric guidelines on a risk cut-off for CRP levels in children. [44] Other studies in CRP in children, however, have applied the adult criteria to define an dichotomous outcome of elevated CRP. [58], [90] Although our choice of cut-off reflects a preference for a criterion-based cutoff, we did examine others to ensure that results were not sensitive to the choice of cut-off. Generally, the effect size (odds ratio) was smaller with a more inclusive definition of elevated CRP (e.g., when defined as the top quartile, CRP levels ≥1.2 mg/L were considered “elevated”); however, the association with neighborhood crime/poverty remained significant.

Results may also be related to unmeasured similarities in families that live in the high poverty/crime neighborhoods. As an example, if children in the different neighborhood conditions were differentially exposed to second-hand smoke, this would not have been controlled for in our analyses. However, studies have not found relationships with second-hand smoke exposure and CRP, after adjustment for adiposity, [60], [69] so this particular example is unlikely to have confounded our results. Furthermore, there may be individual level factors not accounted for in the analysis, like smoking and alcohol consumption. Although we did assess both self-reported smoking and alcohol consumption, only 4 children (1.0%) reported smoking and only 5 children (1.3%) reported alcohol consumption. While neither behavior was associated with elevated CRP in our sample, we recognize that these behaviors may have been underreported and may represent unmeasured confounders of the relationship being investigated.

Lastly, although we confirmed a significant relationship between CRP and neighborhood poverty/crime, we did not include any direct psychological measures of stress; therefore, these results cannot confirm the existence of a neighborhood environment-psychological stress-CRP pathway.

In conclusion, children living in neighborhoods with high levels of poverty or crime had elevated CRP levels compared to children from neighborhoods with lower poverty and crime. This result is consistent with a psychosocial pathway favoring the early development of cardiovascular risk that involves chronic stress from exposure to social and physical disorder characteristic of impoverished neighborhoods. Cardiovascular disease pathways involving neighborhood stress may initiate in childhood. Thus, prevention and early disease screening may have maximal impact when targeting children living in neighborhoods with high levels of poverty or crime.
